# Investigating evidence for a causal association between inflammation and self-harm: A multivariable Mendelian Randomisation study

**DOI:** 10.1016/j.bbi.2020.05.065

**Published:** 2020-10

**Authors:** Abigail Emma Russell, Tamsin Ford, David Gunnell, Jon Heron, Carol Joinson, Paul Moran, Caroline Relton, Matthew Suderman, Gibran Hemani, Becky Mars

**Affiliations:** a Centre for Academic Mental Health, Population Health Sciences, University of Bristol Medical School, United Kingdom; b University of Cambridge, Department of Psychiatry, United Kingdom; c NIHR Biomedical Research Centre at the University Hospitals Bristol NHS Foundation Trust and the University of Bristol, United Kingdom; d MRC Integrative Epidemiology Unit, University of Bristol Medical School, Population Health Sciences, University of Bristol Medical School, United Kingdom

**Keywords:** C-reactive protein, Self-harm, Suicide, Interleukin-6, Inflammation, Mendelian Randomisation

## Abstract

**Background:**

The causal role of inflammatory markers on self-harm and suicidal risk has been studied using observational data, with conflicting results. Confounding and reverse causation can lead to bias, so we appraised question from a genetic perspective to protect against these biases. We measured associations between genetic liability for high levels of inflammatory markers Interleukin-6 (IL-6) and C-reactive protein (CRP) on self-harm, and conducted a secondary analysis restricted to self-harm with suicidal intent.

**Methods:**

We conducted two sample and multivariable Mendelian randomisation (MR) to assess the effects of IL-6 and CRP on self-harm utilising existing data and conducting new genome wide association studies to instrument IL-6 and CRP, and for the outcome of self-harm.

**Results:**

No single nucleotide polymorphisms (SNPs) reached genome-wide significance for self-harm, however 193 SNPs met suggestive significance levels (p < 5 × 10^−6^). We found no evidence of an association between our instruments for IL-6 and self-harm in the two-sample MR, however we found an inverse association between instruments for CRP and self-harm, indicating that higher levels of circulating CRP may protect against self-harm (inverse variance weighted OR 0.92, 95%CI 0.84, 1.01, p = 0.08; MR Egger OR 0.86, 95% CI 0.74, 1.00, p = 0.05). The direct effect estimate for IL-6 was slightly smaller in the multivariable MR than in the two sample MR, while the CRP effect estimates were consistent with the two sample MR (OR 0.92, SE 1.05, p = 0.09).

**Conclusions:**

Our findings are conflicting and indicate that IL-6 and CRP are not robust etiological markers of increased self-harm or suicide risk.

## Highlights

• Observational studies of the role of inflammation on self-harm have conflicting results.
• We used Mendelian Randomisation, a novel causal inference technique to explore this.
• Genetic liability for high levels of IL-6 were not associated with self-harm.
• We found some evidence that higher levels of CRP were protective for self-harm.
• This potential protective effect of CRP has also been found for schizophrenia.

## 1 Introduction

Self-harm and suicide are global public health priorities (World Health Organization, 2012), however we do not currently have a sufficient understanding of the aetiology of these behaviours to reliably identify those at risk. Identifying causal influences of self-harm and suicide risk is an important avenue of research as it may lead to novel prevention or intervention efforts. One mechanism of interest is inflammation, given the emerging literature evidencing associations between altered inflammatory profiles and suicidal behavior (Serafini et al., 2020). Suicidal behaviour is also strongly associated with depression (although does not occur solely in the context of depression), and the two may share common aetiological pathways, or depression may partially mediate associations between inflammatory profiles and suicidal behaviour. There are established links between inflammation and depression, and depression and suicidal behaviour (Haapakoski et al., 2015, Konsman, 2019), however evidence also suggests that inflammation may be associated with suicidal behaviour independently of depression or other psychiatric disorders (Del Giudice and Gangestad, 2018, Huang et al., 2019). It is therefore currently unknown whether inflammation causally impacts on risk of self-harm or suicide in the general population. Self-harm is one of the strongest risk factors for suicide attempt and completed suicide. Understanding the causal antecedents of self-harm may help to identify those at risk of later suicidal behaviour, as well as aid in development of novel treatment and prevention approaches. Inflammation can be broadly categorised in two ways: *systemic inflammation* refers to low grade activation of the immune system, detected through measuring levels of circulating inflammatory molecules, cells and cytokines (signalling molecules secreted by cells related to the immune response) in the bloodstream (Konsman, 2019, Del Giudice and Gangestad, 2018). Alternately the acute *immune response* to bacterial or other challenges can be measured by changes in levels of inflammatory markers following an immune challenge.

The precise mechanisms underlying the relationship between inflammation, mood and risk of self-harm or suicide are currently unknown, and the direction of causality between inflammation and mood is still unclear. Much more research is needed to identify the specific biological pathways that precipitate suicidal behaviour; identifying causal associations between inflammatory antecedents and later suicidal behaviour may help to identify candidate biological pathways (Huang et al., 2019), and advance our understanding of the nature of the relationship between inflammatory markers and suicidal behaviour. Various inflammatory molecules cross the blood–brain barrier, conveying signals to the central nervous system. Some neurons also have receptors for inflammatory cytokines: it is plausible that inflammation could have specific and direct effects on neuronal functioning, potentially impacting on levels of neurotransmitters such as serotonin, leading to altered mood and behaviour (Brundin et al., 2015). An alternate mechanism of effect is via the kynurenine pathway, whereby the cascade of enzymatic effects caused by inflammation ultimately impacts on neuroinflammation, glutamate neurotransmission and the production of serotonin. There is some evidence for altered activation of this pathway in participants with suicidal ideation or attempt (Brundin et al., 2015).

Studies have reported associations between higher levels of circulating inflammatory markers (systemic inflammation) and self-harm, suicide and suicide attempt (Serafini et al., 2020, Black and Miller, 2015, Brundin et al., 2017, Osimo et al., 2018, Ganança et al., 2016). Studies examining inflammatory correlates of suicidal behaviour often focus on clinical samples of participants with psychiatric disorders, or post-mortem studies of individuals who have died by suicide (Osimo et al., 2018, Courtet et al., 2015, Ekinci and Ekinci, 2017, Kim et al., 2014, Keaton et al., 2019, Serafini et al., 2013). Findings from epidemiological studies utilising representative non-clinical samples are inconclusive; some have detected associations between C-reactive protein (CRP) and suicidal behaviour (Batty et al., 2016, Batty et al., 2018), while others fail to identify this association independent of psychiatric disorder (Bergmans et al., 2019, Russell et al., 2019). Findings in studies that focus on self-harm are also mixed: one study based on Danish population registers reported no link between psoriasis (an inflammatory skin disease) and clinical records of self-harm (likely to be predominantly suicidal self-harm) (Egeberg et al., 2016), however another register-based study of English patients found a higher risk of self-harm in individuals with inflammation-related conditions including asthma, psoriasis and inflammatory polyarthropathies (Singhal et al., 2014).

In spite of this mixed evidence, a recent systematic review of associations between inflammatory cytokines and suicidal behaviour concluded that most studies reported associations between high levels of pro-inflammatory cytokines and suicidal behaviour, although they were not able to assess whether there was a causal link between the two (Serafini et al., 2020, Serafini et al., 2013). We recently explored observational associations between levels of interleukin-6 (IL-6) and CRP in childhood and later self-harm in the Avon Longitudinal Study of Parents and Children (ALSPAC) cohort. We detected a slightly higher risk of self-harm in those with higher levels of IL-6, but not CRP. Our design was prospective, however other studies reporting associations between IL-6, CRP and suicidal behaviour are often cross-sectional and reverse causation cannot be ruled out (Russell et al., 2019).

One causal inference method that minimises the potential influence of confounding factors and reverse causality is Mendelian Randomisation (MR). MR is a form of instrumental variable analysis that utilises genetic data, capitalising on Mendel’s principles that genes are randomly assorted in the population (Haycock et al., 2016) Using genetic association data to investigate epidemiological associations therefore reduces the potential of effects being due to confounding. Using MR, we can examine whether genetic markers that confer higher levels of IL-6 and CRP are associated with the risk of self-harm, thus inferring whether there is likely to be a causal relationship from inflammation to self-harm. Similar methods have been used to study the association between inflammation and other psychiatric outcomes such as schizophrenia and depression (Hartwig et al., 2017, Prins et al., 2016, Khandaker et al., 2018).

In order to conduct MR analysis on self-harm, genome-wide association data is required. Three genome-wide association studies (GWASs) of suicidal behaviour have been conducted (Erlangsen et al., 2018, Strawbridge et al., 2019, Mullins et al., 2019): two of these are confined to suicide attempt in populations with psychiatric disorder, and may be susceptible to collider bias due to conditioning on case status (Paternoster and Tilling, 2017). The third examined a spectrum of suicidal behaviour outcomes (including suicidal thoughts) in UK Biobank using ordinal regression, assuming that these behaviours lie on a continuum of severity (Strawbridge et al., 2019) although evidence supporting the latter is contested (Kapur and Cooper, 2013, Mars et al., 2014). As these existing data were not suitable for our research questions, we conducted separate GWASs of self-harm (regardless of suicidal intent), and suicide attempt in UK Biobank as dichotomous outcomes.

In the current study we focus on chronic systemic inflammation, indexed by two well-studied inflammatory markers: IL-6, a largely pro-inflammatory cytokine, and CRP, an acute-phase protein released by the liver in response to levels of inflammatory cytokines including IL-6 (Del Giudice and Gangestad, 2018). We utilised genetic epidemiological data to investigate the potential causal role of two agents that indicate systemic inflammation in the aetiology of self-harm and suicide attempt, and estimate the magnitude of these effects.

## 2 Methods and materials

### 2.1 Study design

**Fig. 1:**
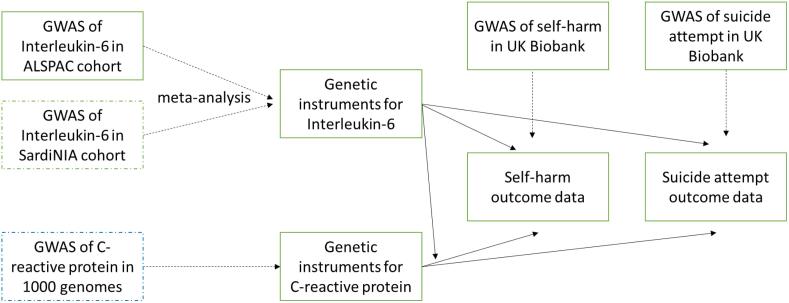
Study data sources. Notes: Dashed arrows represent data sources. Solid arrows represent analyses. Dashed-outlined boxes represent existing data sources shared by their authors, solid outlined boxes represent new GWASs conducted for the current study. GWAS genome-wide association study. ALSPAC the Avon Longitudinal Study of Parents and Children.

A two-sample MR design was used to assess whether levels of IL-6 and CRP are associated with risk of self-harm. Two sample MR utilizes genome-wide summary data to instrument IL-6 and CRP, identifying those with a high genetic risk for high circulating levels of these inflammatory markers; and investigates their association with self-harm in a separate sample. There are three assumptions that underlie MR: firstly that the genetic instrument is a robust predictor of the exposure; secondly that the instrument is independent of confounders of the exposure-outcome relationship; and thirdly that the instrument is only associated with the outcome through the exposure i.e. it has no pleiotropic effects (Supplementary Fig. 1) (Haycock et al., 2016). As production of IL-6 triggers CRP, this third assumption would not hold true for IL-6 unless we include CRP within the same model: thus we conducted separate two-sample MRs of IL-6 and CRP with self-harm, and then a multivariable MR of both IL-6 and CRP with self-harm. Prior to conducting the MR, we conducted genome-wide association studies for IL-6 and self-harm, and meta-analysed our IL-6 GWAS with an existing dataset (the SardiNIA cohort). Fig. 1 illustrates the data sources for our analysis.

### 2.2 Measures

#### 2.2.1 Exposure: Instruments for CRP

Instruments for CRP were derived from the summary associations reported by the 1000 Genomes GWAS for circulating levels of CRP (n = 48,164), described in full elsewhere (Ligthart et al., 2018). 8002 single nucleotide polymorphisms (SNPs) exceeded genome-wide significance thresholds. To ensure instruments were independent, strict clumping was performed with an R^2^ threshold of 0.001 using the *clump* command in the TwoSampleMR package in R, which uses European samples in the 1000 genomes project reference dataset as a reference panel for linkage disequilibrium (LD) (Hemani et al., 2018). Following clumping, 48 SNPs remained and were utilised as instruments for CRP (Supplementary Table 3).

#### 2.2.2 Exposure: Instruments for IL-6

Existing genome-wide association studies of IL-6 have small sample sizes. In order to obtain the largest sample size for our instrument, we conducted a GWAS of IL-6 in the ALSPAC cohort (n = 3,675; see Supplementary material) and meta-analysed our results with those of an existing GWAS of IL-6 in 4,621 Sardinian adults (Naitza et al., 2012).

#### 2.2.3 Meta-analysis of IL-6 GWAS

Meta-analysis of the two GWAS datasets was conducted using METAL, assigning weights to effect size estimates by using the inverse standard errors (Willer et al., 2010). As IL-6 is causal for CRP, and the CRP GWAS is substantially larger than the IL-6 meta-analysis that we performed, we hypothesised that some of the CRP hits may be IL-6 variants that we were unable to detect in our IL-6 meta-analysis. In an attempt to enrich our IL-6 instruments, we evaluated whether any of the CRP instruments described below were predictive of IL-6 at a p-value adjusted for 48 multiple tests (the number of CRP instruments). In additional, post-hoc analyses, we identified 12 SNPs previously-associated with IL-6 from other studies (rs4556348, rs1542176, rs6743376, rs73026617, rs11264224, rs34693607, rs2228145, rs7529229, rs12740969, rs12083537, rs12059682, rs4845371) (Hartwig et al., 2017, Khandaker et al., 2019, Georgakis et al., 2019). We extracted these SNPs from our meta-analysis, performed clumping and used the remaining 3 SNPs in a Two Sample MR of IL-6 and self-harm.

#### 2.2.4 Outcome: self-harm

##### 2.2.4.1 GWAS of self-harm in UK Biobank

As detailed in the introduction, no GWASs of self-harm as a dichotomous outcome exist, however there have been several on a range of suicidal behaviours, most commonly suicide attempt. Another limitation of existing studies is that they use clinical populations, restricted to those with psychopathology. For the genome-wide association analysis of self-harm, we utilised data from UK Biobank, a population-based health research resource consisting of approximately 500,000 people, aged between 38 years and 73 years, who were recruited between the years 2006 and 2010 from across the UK (Allen et al., 2014). A full description of the study design, participants and quality control (QC) methods have been described in detail previously (Collins, 2012). UK Biobank received ethical approval from the North West Multi-centre Research Ethics Committee.

##### 2.2.4.2 Self-harm and suicide attempt

Data on self-harm were collected in an online mental health self-assessment questionnaire in 2016: 48% of invited Biobank participants had completed this by October 2017. Participants were asked “Have you ever deliberately harmed yourself, whether or not you meant to end your life?” Data were available from 153,560 individuals with 4.37% indicating that they had ever self-harmed. Self-harm was utilised as a dichotomous outcome (yes/no) in the GWAS. Suicide attempt was determined from response to the question ““Have you harmed yourself with the intention to end your life?” 2.26% of participants indicated that they had previously self-harmed with suicidal intent (52% of those who reported self-harm).

##### 2.2.4.3 Genotyping and imputation, data quality control

These are described in the supplementary methods. Quality Control filtering of the UK Biobank data for the current study was conducted as described in the protocol described at doi:https://doi.org//10.5523/bris.1ovaau5sxunp2cv8rcy88688v (Mitchell et al., 2017, Bycroft et al., 2018, Howie et al., 2011, Ruth Mitchell et al., 2019). We restricted the sample to individuals of ‘European’ ancestry as defined by an in-house k-means cluster analysis performed using the first four principal components provided by UK Biobank in the statistical software environment R.

##### 2.2.4.4 Genome wide association analysis

GWAS was conducted using linear mixed model (LMM) association method as implemented in BOLT-LMM (v2.3) (Loh et al., 2015). To model population structure in the sample we used 143,006 directly genotyped SNPs, obtained after filtering on MAF > 0.01; genotyping rate > 0.015; Hardy-Weinberg equilibrium p-value < 0.0001 and LD pruning to an r2 threshold of 0.1 using PLINKv2.00. Because the case fraction for suicide attempt was low, suicide attempt GWAS data were filtered on MAF > 0.1 (Loh et al., 2018). Genotype array and sex were adjusted for in the model. Test statistics (betas and their corresponding standard errors) were transformed to log odds ratios and their corresponding 95% confidence intervals on the liability scale using a Taylor transformation expansion series (Loh et al., 2017).

##### 2.2.4.5 Mendelian randomisation analysis

In order to estimate the total effect of IL-6 and CRP individually on self-harm, two sample MR was performed for each exposure using the TwoSampleMR package in R (RC Team, 2013). The inverse variance weighting (IVW) method was used as the primary analysis, which calculates the inverse variance weighted mean of ratio estimates from the instruments (i.e. the SNP IL-6 effect estimate divided by the SNP self-harm effect estimate). An important limitation in MR analysis is that it is difficult to prove that there is no path from the instrument to the outcome that is not through the exposure (also known as horizontal pleiotropy (Hemani et al., 2018). We used ‘pleiotropy robust’ methods as sensitivity analysis in addition to the main IVW approach. These included MR Egger regression: a weighted linear regression of the outcome against exposure SNP estimates (Bowden and Davey Smith, 2015), and the simple mode, weighted mode and weighted median methods for the CRP MR as sensitivity analyses to explore whether our findings were consistent across different models of horizontal pleiotropy. Cochran’s Q tests were used to assess heterogeneity of the IVW estimates, which is a global measure of horizontal pleiotropy. Global directional pleiotropy, which would indicate if pleiotropy was generally leading to bias, was assessed through funnel plots and through evaluating the magnitude of the intercept from MR Egger regression for CRP. If this formal statistical test of directional pleiotropy is significantly different from zero, this implies the presence of a pathway between the instruments and outcome that is not mediated by the exposure.

To estimate the effects of IL-6 and CRP on self-harm simultaneously, multivariable MR was conducted in R using the IVW and MR Egger methods. This provides causal effect estimates for each biomarker, conditioning on the other. Therefore, estimates from the two sample MRs can be considered analogous to the total effect in an epidemiological mediation model, and the multivariable MR estimates are the direct effect of each biomarker on the outcome. To conduct the multivariable MR, all genetic instruments for IL-6 and CRP were extracted from both the IL-6 meta-analysis and the CRP GWAS. These were harmonised and clumped as above using the TwoSampleMR package in R. The IVW and MR Egger effect estimates were calculated for IL-6 and CRP on the outcome of self-harm, adjusting for the effect operating through the other inflammatory marker. Potential global pleiotropy was assessed through examining consistency of the causal effect estimates, and F statistics were generated to assess weak instrument bias (Hartwig et al., 2017, Khandaker et al., 2019, Georgakis et al., 2019, Staiger and Stock, 1994).

## 3 Results

### 3.1 Self-harm and suicide attempt GWAS’s in UK Biobank

**Fig. 2:**
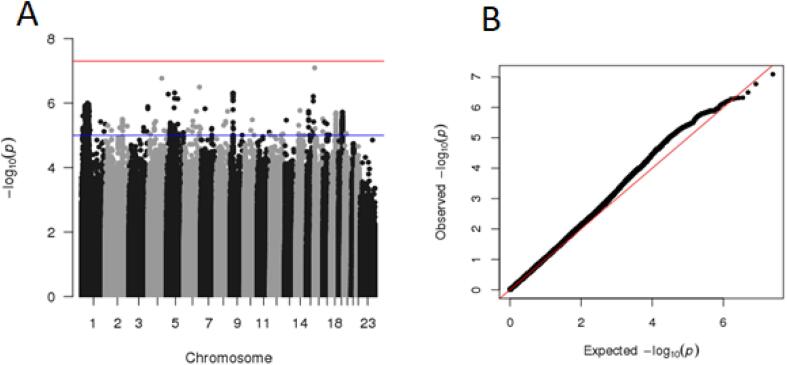
A) Manhattan plot and B) quantile–quantile plot of self-harm genome wide association study results in UK Biobank.

There were no SNPs that were statistically significantly associated with self-harm in our GWAS (at p < 5 × 10^−8^), however 193 SNPs met suggestive significance levels at p < 5 × 10^−6^ with the majority being on chromosome 1 and 19 (Fig. 2). The ‘suggestive’ significance threshold represents one false positive result being expected per GWAS, as proposed by Lander and Kruglyak (Lander, 1995) Similarly, there were no SNPs that met genome-wide significance thresholds in the suicide attempt GWAS, 15 met suggestive thresholds (Supplementary Fig. 2; Supplementary Table 2).

### 3.2 IL-6 GWAS and meta-analysis

**Fig. 3:**
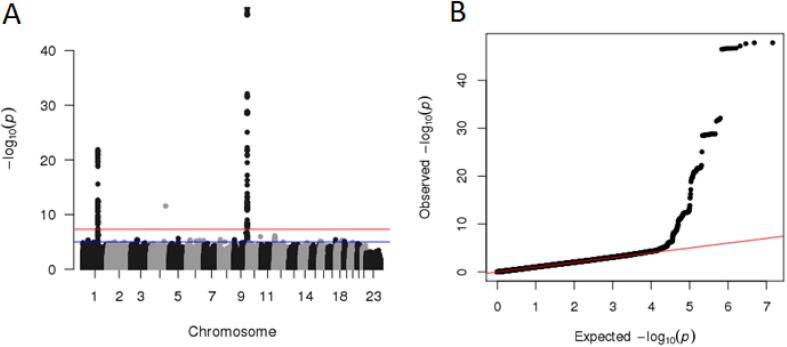
A) Manhattan plot and B) quantile–quantile plot of Interleukin-6 Meta-analysis of ALSPAC and SardiNIA cohorts.

In the GWAS of IL-6 in ALSPAC 190 single nucleotide polymorphisms (SNPs) on chromosomes 1 and 9 reached genome-wide significance (p < 5 × 10^−8^). The meta-analysis of the SardiNIA and ALSPAC IL-6 data resulted in 198 genome-wide significant hits from a total sample of 8296 individuals (Fig. 3). Following clumping as described above, this resulted in two genome-wide significant hits (rs56383622 on chromosome 1, and rs643434 on chromosome 9) that we utilised as instruments for IL-6 in the MR. Two further SNPs (rs2228145 on chromosome 1 and rs505922 on chromosome 9) from the CRP instruments were associated with IL-6 levels, however these were in LD with our existing instruments and were subsequently excluded during clumping (Supplementary Table 4).

### 3.3 Estimates of the total effects of IL-6 and CRP on self-harm- two sample MR

Effect estimates from the two sample MR of IL-6 and self-harm were null, indicating no evidence of an association between our IL-6 instruments and self-harm (Table 1). In the follow-up post-hoc analysis we assessed the 12 SNPs previously associated with IL-6 in Hartwig et al., 2017, Khandaker et al., 2019, Georgakis et al., 2019: Following clumping, 3 SNPs remained (see Supplementary Table 5; rs1542176, rs6743376, rs7529229) however only rs7529229 was significantly associated with IL-6 in our meta-analysis (p = 4.15 × 10^−22^) with p > 0.2 for the other 2 SNPs. We entered these 3 SNPs as instruments into a further 2-sample MR of IL-6 against self-harm. The IVW results showed no association between these instruments and self-harm (OR 1.00, 95% CI 0.84, 1.19), and results were consistently null for the instruments when assessed individually.

**Table 1:** Two sample MR results.

	Interleukin-6 (n SNPs = 2)			C-reactive protein (n SNPs = 47)		
Model	OR	95% CI	p	OR	95% CI	p
IVW	1.14	0.90, 1.45	0.28	0.92	0.84, 1.01	0.08
MR Egger				0.86	0.74, 1.00	0.05
Weighted median				0.88	0.78, 1.00	0.05
Simple mode				0.96	0.77, 1.21	0.76
Weighted mode				0.88	0.79, 0.98	0.02

*Notes*: SNP single nucleotide polymorphism (genetic instruments) IVW inverse variance weighted, OR odds ratio CI confidence interval.

We found an inverse association between CRP and self-harm, indicating that higher levels of circulating CRP are protective against self-harm (IVW OR 0.92, 95%CI 0.84, 1.01, p = 0.08; MR Egger OR 0.86, 95% CI 0.74, 1.00, p = 0.05). Cochran’s Q test indicated no apparent heterogeneity in the inverse variance estimates from each genetic variant individually (IL-6 Q(1) = 0.21, p = 0.65; CRP Q(46) = 1.08, p = 0.52) (Bowden and Davey Smith, 2015), and tests for pleiotropy in the CRP instruments showed that the Egger intercept was not significantly different from zero, indicating that directional pleiotropy is unlikely (CRP intercept 0.01 SE 0.01p = 0.24). For CRP, we also calculated estimates based on the weighted median and weighted mode: methods that are more robust to pleiotropy. These methods were not suitable for IL-6 as we had only two instruments. For CRP, the effect estimates and confidence intervals were consistent with the IVW and MR Egger findings, indicating that higher CRP levels may be protective against self-harm.

### 3.4 Estimates of the direct effects of IL-6 and CRP on self-harm: Multivariable MR

We found no evidence of an association between IL-6 and self-harm in the multivariable MR (Table 2). The direct effect estimate for IL-6 was slightly smaller in the IVW multivariable MR than in the 2-sample MR (OR reduced from 1.14 to 1.09), and the CRP effect estimates were consistent with the 2-sample MR, supporting the finding of a reduced risk of self-harm with increasing levels of CRP, although in the MVMR this was no longer statistically significant (OR 0.92, SE 1.05, p = 0.09). The MR Egger intercept of the MVMR was not large and not significantly different from zero (intercept = −0.002, SE = 0.005, p = 0.74), and the causal effect estimates were again largely consistent suggesting that additional pleiotropy is not meaningfully biasing the results. The F statistics were much lower than the rule-of-thumb cut-off of 10 (IVW F = 2.33, p = 0.11, Egger F = 2.12, p = 0.13) indicating weak instrument bias (Sanderson et al., 2018).

**Table 2:** Two sample MR and MVMR results.

		Two sample MR (total effects)			Multivariable MR (direct effects) n SNPs = 46		
Method	Biomarker	OR	95% CI	p	OR	SE	p
IVW	IL-6	1.14	(0.90, 1.45)	0.28	1.09	1.10	0.37
	CRP	0.92	(0.84, 1.01)	0.08	0.92	1.05	0.09
Egger	IL6				1.11	1.12	0.36
	CRP	0.86	(0.74, 1.00)	0.05	0.92	1.05	0.09

*Notes*: IVW: inverse variance weighted; IL-6 Interleukin-6, CRP C-reactive protein. OR odds ratio 95% CI 95% confidence interval, SE standard error. In two sample MR the effects of IL-6 on self-harm, and CRP on self-harm, are estimated in separate models. In multivariable MR IL-6 and CRP are modelled simultaneously, being conditioned on each other.

### 3.5 Secondary analysis: Suicide attempt outcome

We conducted a secondary analysis to explore whether our findings were consistent with a more specific definition of self-harm with suicidal intent (Table 3, Table 4). In the two-sample MR of IL-6 on suicide attempt, lower IL-6 was associated with an increased risk of suicide attempt, although the confidence intervals overlapped the null value substantially as in the self-harm MR. In the two-sample MR of CRP on suicide attempt, higher CRP was associated with an increased risk of suicide attempt, however the confidence intervals were much wider and overlapped with the null value and the confidence intervals in the self-harm analysis. Findings were consistent across different MR methods. In the multivariable MR, the effect estimates for both IL-6 and CRP were null (Table 3, Table 4).

**Table 3:** Two sample MR – suicide attempt.

	Interleukin-6 (n SNPs = 2)			C-reactive protein (n SNPs = 47)		
Model	OR	95% CI	p	OR	95% CI	p
IVW	0.83	(0.38, 1.81)	0.64	1.24	(0.81, 1.91)	0.33
MR Egger				1.60	(0.82, 3.14)	0.18
Weighted median				1.31	(0.70, 2.47)	0.40
Simple mode				1.08	(0.32, 3.66)	0.90
Weighted mode				1.44	(0.83, 2.49)	0.20

*Notes*: IVW inverse variance weighted, OR odds ratio CI confidence interval, SNP single nucleotide polymorphism (genetic instrument).

**Table 4:** Multivariable MR results – suicide attempt.

		Two sample MR (total effects)			Multivariable MR (direct effects) n SNPs = 46		
Method	Biomarker	OR	95% CI	p	OR	SE	p
IVW	IL-6	0.83	(0.38, 1.81)	0.64	0.89	1.23	0.57
	CRP	1.24	(0.81, 1.91)	0.32	1.25	1.23	0.28
Egger	IL-6				1.04	1.26	0.88
	CRP	1.60	(0.82, 3.14)	0.18	1.25	1.22	0.28

*Notes*: IVW: inverse variance weighted; IL-6 Interleukin-6, CRP C-reactive protein. OR odds ratio 95% CI 95% confidence interval, SE standard error, SNP single nucleotide polymorphism (genetic instrument). In two sample MR the effects of IL-6 on self-harm, and CRP on self-harm, are estimated in separate models. In multivariable MR IL-6 and CRP are modelled simultaneously, being conditioned on each other.

## 4 Discussion

In our two-sample MR analyses we found no evidence of an association between IL-6 and self-harm, and some evidence that higher levels of CRP may be protective against self-harm. Our findings suggest conflicting evidence of a causal role of these two inflammatory agents in the aetiology of self-harm, suggesting that different inflammatory markers may have differential or no associations with self-harm in European populations.

A major limitation of existing observational studies in this field is the use of clinical samples, i.e. individuals with diagnosed psychiatric illnesses. Although self-harm and suicide is more common in those with psychiatric disorders (Singhal et al., 2014), the majority of those who self-harm will not have a psychiatric diagnosis or attempt suicide (Mars et al., 2014). Associations between inflammation and self-harm, suicide attempt or suicide in psychiatric populations may be either confounded by or unique to the disorder being studied. Similarly, existing GWASs of self-harm and suicide-related behaviours are also confined to samples that have high proportions of individuals with diagnosed psychiatric disorders (Erlangsen et al., 2018, Mullins et al., 2019), with the exception of the current study and the recently published study using the same UK Biobank sample (Strawbridge et al., 2019). Our finding of no genome-wide significant associations for self-harm and suicide attempt may indicate that these behaviours do not have a strong underlying genetic basis, which would limit our ability to detect associations with IL-6 and CRP in our outcome genetic dataset relating to self-harm risk, or may be due to a cohort effect.

Both IL-6 and CRP are commonly used as biomarkers of inflammation. However, the mechanisms and actions of these molecules are complex. IL-6 is a family of ten cytokines that operate via two pathways and has pro- and anti-inflammatory effects: the classic signalling pathway which increases CRP production, and the *trans*-signalling pathway which is upregulated during inflammation (Konsman, 2019, Del Giudice and Gangestad, 2018, Murakami et al., 2019). Therefore although IL-6 acts in a pro-inflammatory manner, it also contributes to reduction of the immune response, tissue repair and regeneration and thus has anti-inflammatory effects (Konsman, 2019, Del Giudice and Gangestad, 2018, Murakami et al., 2019). CRP also has varied effects, including binding to damaged cells during the inflammatory response, activating neutrophils and monocytes and promoting the secretion of inflammatory cytokines. It also has anti-inflammatory effects. The form of CRP released by the liver, and detectable as circulating CRP, has mainly anti-inflammatory effects, but when this encounters other signals of inflammation or cell damage it breaks down into five identical molecules that are pro-inflammatory in nature (Del Giudice and Gangestad, 2018, Black and Kushner, 2004). Therefore, although elevated IL-6 and CRP may be due to inflammation, they can exist in the absence of an inflammatory state and do not *define* an inflammatory state (Konsman, 2019, Del Giudice and Gangestad, 2018), using these as markers of systemic inflammation may even be misleading or inaccurate. Further studies are needed to examine the role of other inflammatory molecules in relation to self-harm to explore whether findings are consistent where molecules have solely pro- or anti-inflammatory effects.

Our findings that higher levels of CRP are associated with reduced risks of self-harm may not necessarily mean that inflammation is protective against self-harm: conversely the anti-inflammatory effect of higher levels of CRP may be acting as a protective mechanism. Other MR studies of CRP have also failed to find robust evidence for higher disease risk with higher circulating CRP levels across a range of physical and mental health outcomes, contrary to other observational findings (Prins et al., 2016). Two studies have however found evidence that higher CRP is protective against schizophrenia, mirroring our finding regarding self-harm (Hartwig et al., 2017, Prins et al., 2016): these authors have hypothesised that this may reflect increased susceptibility to early-life infection, although it may also be influenced by socioeconomic or lifestyle characteristics (Hartwig et al., 2017). Equally, we also concede the possibility that the detected association may have arisen by chance, and our findings need replicating before further conclusions can be drawn.

Links between inflammation and self-harm reported by existing observational studies may be due to reverse causality i.e. self-harm causing inflammation, or confounding, for example by the presence of chronic and painful diseases that are associated with both suicide risk and inflammation (Singhal et al., 2014). Indeed, not all studies of the relationship between IL-6 and suicidal behaviours find associations (Ganança et al., 2016). Compared with observational studies using conventional regression methods, MR studies are less likely to be affected by reverse causality and reduce bias due to unmeasured confounding.

### 4.1 Strengths and limitations

The instruments we used for CRP were generated from a large sample size, and genetic instruments for CRP have been shown to be strong, reflecting up to 5% of variation in measured CRP levels (Ligthart et al., 2018). However, instruments for IL-6 are less well established. Only two existing GWAS studies of IL-6 had been published prior to the current study (Naitza et al., 2012, Ahola-Olli et al., 2017), one of which we meta-analysed with our GWAS in the ALSPAC cohort in order to increase power and sample size. Despite this, our total sample was still under 10,000. Because of the limited number of instruments for IL-6 and our relatively small sample size (in genetic epidemiology terms), we had limited power to detect effects of IL-6 in our MR. The samples for our exposures and outcomes in the current study were restricted to those of European ancestry, limiting the possibility of population stratification bias, but further reducing sample size. We explored the influences of two inflammatory markers that have previously been shown to be associated with self-harm and suicidal behaviour, however in a biological pathway leading to a complex outcome such as self-harm many molecules and agents will be implicated, so we are limited in what can be inferred from examining only two of these markers.

A strength of our study was that we are the first to assess relationships between inflammation and self-harm using genetically-informed methods. Our GWAS of self-harm overcomes limitations of existing GWASs of suicidal behaviour as the sample was non-clinical, and most cases of self-harm do not come to clinical attention (Hawton et al., 2012). Existing GWASs of suicidal behaviour focus on suicide attempt ascertained from clinical records, subject to case identification bias as only around half of individuals who attempt suicide present to services (McManus et al., 2016), and we have expanded currently limited knowledge about the genetic architecture of self-harm. However, our study had limitations regarding the generalisability of our GWAS findings. Although we had a large sample size of ~150,000 individuals from UK Biobank, the prevalence of self-harm (4.4%) was lower than expected given the prevalence of lifetime non-suicidal self-harm of 7.3% and suicide attempt of 6.7% in UK population-representative data (McManus et al., 2016). Participants in UK Biobank are not representative of the UK population: they are of higher SES and more physically and mentally healthy, and both rates and reporting of self-harm may be biased (Fry et al., 2017). There is a need for further studies using representative population samples to collect data on self-harm and other suicidal behaviours in addition to genetic data in order to increase our understanding of the genetic architecture of these traits beyond psychiatric populations.

## 5 Conclusion

This is this first study of which we are aware that investigates potential causal associations between levels of two commonly-measured inflammatory markers and self-harm, using genetic epidemiological methods. Our findings are conflicting and indicate that the inflammatory markers IL-6 and CRP are not robust aetiological markers of increased risk of self-harm or suicide attempt. Individual inflammatory molecules may have differential associations with self-harm, and further research should aim to pinpoint hypothesised mechanisms of action between inflammatory molecules and risk of self-harm or suicide.

## Declaration of Competing Interest

The authors declare that they have no known competing financial interests or personal relationships that could have appeared to influence the work reported in this paper.

## References

[b0005] Organization WH. Public health action for the prevention of suicide: a framework. 2012.

[b0010] 2. Serafini G, Parisi VM, Aguglia A, Amerio A, Sampogna G, Fiorillo A, et al. A specific inflammatory profile underlying suicide risk? Systematic review of the main literature findings. 2020;17(7):2393. 10.3390/ijerph17072393 PMC7177217 32244611

[b0015] Haapakoski R., Mathieu J., Ebmeier K.P., Alenius H., Kivimäki M. (2015). Cumulative meta-analysis of interleukins 6 and 1β, tumour necrosis factor α and C-reactive protein in patients with major depressive disorder. Brain Behav. Immun..

[b0020] Konsman JPJP. Inflammation and depression: a nervous plea for psychiatry to not become immune to interpretation. 2019;12(1):29. 10.3390/ph12010029 PMC6469164 30769887

[b0025] Del Giudice M., Gangestad S.W. (2018). Rethinking IL-6 and CRP: Why they are more than inflammatory biomarkers, and why it matters. Brain Behav Immun..

[b0030] Huang M., Su S., Goldberg J., Miller A.H., Levantsevych O.M., Shallenberger L. (2019). Longitudinal association of inflammation with depressive symptoms: A 7-year cross-lagged twin difference study. Brain Behav Immun..

[b0035] Brundin L., Erhardt S., Bryleva E., Achtyes E.D., Postolache T. (2015). The role of inflammation in suicidal behaviour. Acta Psychiatr. Scand..

[b0040] Black C., Miller B.J. (2015). Meta-analysis of cytokines and chemokines in suicidality: distinguishing suicidal versus nonsuicidal patients. Biol. Psychiatry.

[b0045] Brundin L., Bryleva E.Y., Rajamani K.T. (2017). Role of inflammation in suicide: from mechanisms to treatment. Neuropsychopharmacology.

[b0050] Osimo E.F., Cardinal R.N., Jones P.B., Khandaker G.M. (2018). Prevalence and correlates of low-grade systemic inflammation in adult psychiatric inpatients: an electronic health record-based study. Psychoneuroendocrinology.

[b0055] Ganança L., Oquendo M.A., Tyrka A.R., Cisneros-Trujillo S., Mann J.J., Sublette M.E. (2016). The role of cytokines in the pathophysiology of suicidal behavior. Psychoneuroendocrinology.

[b0060] Courtet P., Jaussent I., Genty C., Dupuy A.M., Guillaume S., Ducasse D. (2015). Increased CRP levels may be a trait marker of suicidal attempt. Eur Neuropsychopharmacol..

[b0065] Ekinci O., Ekinci A. (2017). The connections among suicidal behavior, lipid profile and low-grade inflammation in patients with major depressive disorder: a specific relationship with the neutrophil-to-lymphocyte ratio. Nord. J. Psychiatry.

[b0070] Kim J.-W., Szigethy E.M., Melhem N.M., Saghafi E.M., Brent D.A. (2014). Inflammatory markers and the pathogenesis of pediatric depression and suicide: a systematic review of the literature. J. Clin. Psychiatry.

[b0075] Keaton S.A., Madaj Z.B., Heilman P., Smart L., Grit J., Gibbons R. (2019). An inflammatory profile linked to increased suicide risk. J. Affect. Disord..

[b0080] Serafini G., Pompili M., Seretti M.E., Stefani H., Palermo M., Coryell W. (2013). The role of inflammatory cytokines in suicidal behavior: a systematic review. Eur. Neuropsychopharmacol..

[b0085] Batty G.D., Bell S., Stamatakis E., Kivimäki M. (2016). Association of systemic inflammation with risk of completed suicide in the general population. JAMA Psychiatry.

[b0090] Batty G.D., Jung K.J., Lee S., Back J.H., Jee S.H. (2018). Systemic inflammation and suicide risk: cohort study of 419 527 Korean men and women. J. Epidemiol Commun. Health.

[b0095] Bergmans R.S., Kelly K.M., Mezuk B. (2019). Inflammation as a unique marker of suicide ideation distinct from depression syndrome among US adults. J. Affect. Disord..

[b0100] Russell AE, Heron J, Gunnell D, Ford T, Hemani G, Joinson C, et al. Pathways between early‐life adversity and adolescent self‐harm: the mediating role of inflammation in the Avon Longitudinal Study of Parents and Children. 2019. 10.1111/jcpp.13100 PMC6771906 31486089

[b0105] Egeberg A., Hansen P.R., Gislason G.H., Skov L., Mallbris L. (2016). Risk of self-harm and nonfatal suicide attempts, and completed suicide in patients with psoriasis: a population-based cohort study. Br. J. Dermatol..

[b0110] Singhal A., Ross J., Seminog O., Hawton K., Goldacre M.J. (2014). Risk of self-harm and suicide in people with specific psychiatric and physical disorders: comparisons between disorders using English national record linkage. J. R. Soc. Med..

[b0115] Haycock P.C., Burgess S., Wade K.H., Bowden J., Relton C., Davey Smith G. (2016). Best (but oft-forgotten) practices: the design, analysis, and interpretation of Mendelian randomization studies. Am. J. Clin. Nutr..

[b0120] Hartwig F.P., Borges M.C., Horta B.L., Bowden J., Smith G.D. (2017). Inflammatory biomarkers and risk of schizophrenia: a 2-sample mendelian randomization study. JAMA Psychiatry.

[b0125] Prins B.P., Abbasi A., Wong A., Vaez A., Nolte I., Franceschini N. (2016). Investigating the causal relationship of C-reactive protein with 32 complex somatic and psychiatric outcomes: a large-scale cross-consortium mendelian randomization study. PLoS Med..

[b0130] Khandaker G.M., Zammit S., Burgess S., Lewis G., Jones P.B. (2018). Association between a functional interleukin 6 receptor genetic variant and risk of depression and psychosis in a population-based birth cohort. Brain Behav. Immun..

[b0135] Erlangsen A., Appadurai V., Wang Y., Turecki G., Mors O., Werge T. (2018). Genetics of suicide attempts in individuals with and without mental disorders: a population-based genome-wide association study. Mol. Psychiatry.

[b0140] Strawbridge R.J., Ward J., Ferguson A., Graham N., Shaw R.J., Cullen B. (2019). Identification of novel genome-wide associations for suicidality in UK Biobank, genetic correlation with psychiatric disorders and polygenic association with completed suicide. EBioMedicine.

[b0145] Mullins N., Bigdeli T.B., Borglum A.D., Coleman J.R.I., Demontis D., Mehta D. (2019). GWAS of suicide attempt in psychiatric disorders and association with major depression polygenic risk scores. Am. J. Psychiatry.

[b0150] Paternoster L, Tilling K, Davey Smith GJPg. Genetic epidemiology and Mendelian randomization for informing disease therapeutics: Conceptual and methodological challenges. 2017;13(10):e1006944. 10.1371/journal.pgen.1006944 PMC5628782 28981501

[b0155] Kapur N, Cooper J, O'connor RC, Hawton K. Non-suicidal self-injury v. attempted suicide: new diagnosis or false dichotomy? The British Journal of Psychiatry. 2013;202(5):326-8. 10.1192/bjp.bp.112.116111 23637107

[b0160] Mars B., Heron J., Crane C., Hawton K., Kidger J., Lewis G. (2014). Differences in risk factors for self-harm with and without suicidal intent: findings from the ALSPAC cohort. J. Affect. Disord..

[b0165] Ligthart S., Vaez A., Vosa U., Stathopoulou M.G., de Vries P.S., Prins B.P. (2018). Genome Analyses of >200,000 individuals identify 58 loci for chronic inflammation and highlight pathways that link inflammation and complex disorders. Am. J. Hum. Genet..

[b0170] Hemani G, Zheng J, Elsworth B, Wade KH, Haberland V, Baird D, et al. The MR-Base platform supports systematic causal inference across the human phenome. 2018;7:e34408. 10.7554/eLife.34408 PMC5976434 29846171

[b0175] Naitza S., Porcu E., Steri M., Taub D.D., Mulas A., Xiao X. (2012). A genome-wide association scan on the levels of markers of inflammation in Sardinians reveals associations that underpin its complex regulation. PLoS Genet..

[b0180] Willer C.J., Li Y., Abecasis G.R. (2010). METAL: fast and efficient meta-analysis of genomewide association scans. Bioinformatics.

[b0185] Khandaker G.M., Zuber V., Rees J.M.B., Carvalho L., Mason A.M., Foley C.N. (2019). Shared mechanisms between coronary heart disease and depression: findings from a large UK general population-based cohort. Mol. Psychiatry.

[b0190] Georgakis MK, Malik R, Gill DK, Franceschini N, Sudlow CL, Dichgans M. Interleukin-6 signaling effects on ischemic stroke and other cardiovascular outcomes: a Mendelian Randomization study. 2019:19007682. 10.1161/CIRCGEN.119.002872 PMC7299212 32397738

[b0195] Allen NE, Sudlow C, Peakman T, Collins R. UK biobank data: come and get it. American Association for the Advancement of Science; 2014. 10.1126/scitranslmed.3008601 24553384

[b0200] Collins R. (2012). What makes UK Biobank special?. Lancet.

[b0205] Mitchell R, Hemani G, Dudding T, Paternoster L. UK biobank genetic data: MRC-IEU quality control, Version 1. University of Bristol. 2017.

[b0210] Bycroft C., Freeman C., Petkova D., Band G., Elliott L.T., Sharp K. (2018). The UK Biobank resource with deep phenotyping and genomic data. Nature.

[b0215] Howie B., Marchini J., Stephens M. (2011). Genotype imputation with thousands of genomes. G3.

[b0220] Ruth Mitchell GH, Tom Dudding, Laura Corbin, Sean Harrison, Lavinia Paternoster. UK Biobank Genetic Data: MRC-IEU Quality Control, version 2. 2019.

[b0225] Loh P.-R., Tucker G., Bulik-Sullivan B.K., Vilhjalmsson B.J., Finucane H.K., Salem R.M. (2015). Efficient Bayesian mixed-model analysis increases association power in large cohorts. Nat. Genet..

[b0230] Loh P-R, Kichaev G, Gazal S, Schoech AP, Price ALJNg. Mixed-model association for biobank-scale datasets. 2018;50(7):906. 10.1038/s41588-018-0144-6 PMC6309610 29892013

[b0235] Loh P-R, Kichaev G, Gazal S, Schoech AP, Price AL. Mixed model association for biobank-scale data sets. bioRxiv. DOI: https://doi org/101101/194944. 2017. 10.1038/s41588-018-0144-6 PMC6309610 29892013

[b0240] Team RC. R: A language and environment for statistical computing. 2013.

[b0245] Hemani G., Bowden J., Davey Smith G. (2018). Evaluating the potential role of pleiotropy in Mendelian randomization studies. Hum. Mol. Genet..

[b0250] Bowden J, Davey Smith G, Burgess SJIjoe. Mendelian randomization with invalid instruments: effect estimation and bias detection through Egger regression. 2015;44(2):512-25. 10.1093/ije/dyv080 PMC4469799 26050253

[b0255] Staiger DO, Stock JH. Instrumental variables regression with weak instruments. National Bureau of Economic Research Cambridge, Mass., USA; 1994.

[b0260] Lander E, Kruglyak LJNg. Genetic dissection of complex traits: guidelines for interpreting and reporting linkage results. 1995;11(3):241. 10.1038/ng1195-241 7581446

[b0265] Sanderson E., Davey Smith G., Windmeijer F., Bowden J. (2018). An examination of multivariable Mendelian randomization in the single-sample and two-sample summary data settings. Int. J. Epidemiol..

[b0270] Murakami M., Kamimura D., Hirano T. (2019). Pleiotropy and Specificity: Insights from the Interleukin 6 Family of Cytokines. Immunity.

[b0275] Black S, Kushner I, Samols DJJoBC. C-reactive protein. 2004;279(47):48487-90. 10.1074/jbc.R400025200 15337754

[b0280] Ahola-Olli A.V., Würtz P., Havulinna A.S., Aalto K., Pitkänen N., Lehtimäki T. (2017). Genome-wide association study identifies 27 loci influencing concentrations of circulating cytokines and growth factors. Am. J. Hum. Genet..

[b0285] Hawton K., Saunders K.E., O'Connor R.C. (2012). Self-harm and suicide in adolescents. Lancet.

[b0290] McManus S, Bebbington P, Jenkins R, Brugha T. Mental Health and Wellbeing in England: Adult Psychiatric Morbidity Survey 2014: a Survey Carried Out for NHS Digital by NatCen Social Research and the Department of Health Sciences, University of Leicester: NHS Digital; 2016.

[b0295] Fry A., Littlejohns T.J., Sudlow C., Doherty N., Adamska L., Sprosen T. (2017). Comparison of sociodemographic and health-related characteristics of UK biobank participants with those of the general population. Am. J. Epidemiol..

