# Supplementary material for: Investigating evidence for a causal association between inflammation and self-harm: A multivariable Mendelian Randomisation study
Source: Brain Behav Immun. 2020 Oct;89:43–50. doi: 10.1016/j.bbi.2020.05.065 (PMC7575900; doi:10.1016/j.bbi.2020.05.065)
Supplement: Supplementary data 1 [file mmc1.docx]

# Supplementary information

## Additional methods

### GWAS of IL-6 in ALSPAC

ALSPAC is a prospective longitudinal birth cohort that has followed 15,454 pregnant mothers who had an expected delivery date between 1/4/1991 to 31/12/1992. 14,901 children were alive at one year of age (1, 2). Please note that the study website contains details of all the data that is available through a fully searchable data dictionary and variable search tool" and reference the following webpage: http://www.bristol.ac.uk/alspac/researchers/our-data/. Ethical approval for the study was obtained from the ALSPAC Ethics and Law Committee and the Local Research Ethics Committees. Consent for biological samples has been collected in accordance with the Human Tissue Act (2004). The sample size comprised all children who attended the “Focus@9” clinic when they were nine years old, had a blood test that was successfully assayed for IL-6, and had genotype data (n=3,675). Blood samples were spun and frozen immediately after collection at -80ᵒC. IL-6 (pg/L) was measured using high sensitivity IL-6 enzyme-linked immunosorbent assay (R&D systems, Abingdon, UK). The intraassay coefficient of variation was less than 5%. These methods have been reported in detail previously (3). IL-6 was natural-logarithm transformed prior to analysis and those reporting recent infection were excluded. Genotyping and quality control are described in the supplementary methods**.** Genotype data were imputed to the HRC panel (HRC.r1.1) using the Michigan imputation server resulting in 8,237 children with genotype data. Related individuals were excluded. A total of 3,675 individuals had both IL-6 and genotype data. The GWAS was conducted using *snptest,* adjusting for age, sex and population substructure by including the first ten principle components. Results were filtered based on minor allele frequency of >0.01 and info score (an indicator of the quality of imputation for each variant) of >0.3.

### ALSPAC genotyping and quality control for Interleukin-6 GWAS

ALSPAC children were genotyped using the Illumina HumanHap550 quad chip genotyping platforms by 23andme subcontracting the Wellcome Trust Sanger Institute, Cambridge, UK and the Laboratory Corporation of America, Burlington, NC, US. The resulting raw genome-wide data were subjected to standard quality control methods. Individuals were excluded on the basis of gender mismatches; minimal or excessive heterozygosity; disproportionate levels of individual missingness (>3%) and insufficient sample replication (IBD < 0.8). Population stratification was assessed by multidimensional scaling analysis and compared with Hapmap II (release 22) European descent (CEU), Han Chinese, Japanese and Yoruba reference populations; all individuals with non-European ancestry were removed. SNPs with a minor allele frequency of < 1%, a call rate of < 95% or evidence for violations of Hardy-Weinberg equilibrium (P < 5E-7) were removed. Cryptic relatedness was measured as proportion of identity by descent (IBD > 0.1). Related subjects that passed all other quality control thresholds were retained during subsequent phasing and imputation. 9,115 subjects and 500,527 SNPs passed these quality control filters. Genotype data were imputed to the HRC panel (HRC.r1.1) using the Michigan imputation server resulting in 8,237 children with genotype data. Related individuals were excluded.

### UK Biobank genotyping and imputation, data quality control

The full data release contains the cohort of successfully genotyped samples (n=488,377). 49,979 individuals were genotyped using the UK BiLEVE array and 438,398 using the UK Biobank axiom array. Quality Control filtering of the UK Biobank data for the current study was conducted as described in the protocol described at doi:[10.5523/bris.1ovaau5sxunp2cv8rcy88688v](https://doi.org/10.5523/bris.1ovaau5sxunp2cv8rcy88688v) (4). Pre-imputation quality control (QC), phasing and imputation are described elsewhere (5). In brief, prior to phasing, multiallelic SNPs or those with MAF ≤1% were removed. Phasing of genotype data was performed using a modified version of the SHAPEIT2 algorithm. Genotype imputation to a reference set combining the UK10K haplotype and HRC reference panels was performed using IMPUTE2 algorithms (6). The analyses presented here were restricted to autosomal variants within the HRC site list using a graded filtering with varying imputation quality for different allele frequency ranges. Therefore, rarer genetic variants are required to have a higher imputation INFO score (Info>0.3 for MAF >3%; Info>0.6 for MAF 1-3%; Info>0.8 for MAF 0.5-1%; Info>0.9 for MAF 0.1-0.5%) with MAF and Info scores having been recalculated on an in-house derived ‘European’ subset (7).

**Data quality control**

Individuals with sex-mismatch (derived by comparing genetic sex and reported sex) or individuals with sex-chromosome aneuploidy were excluded from the analysis (n=814). We restricted the sample to individuals of ‘European’ ancestry as defined by an in-house k-means cluster analysis performed using the first four principal components provided by UK Biobank in the statistical software environment R. The current analysis includes the largest cluster from this analysis (n=464,708) (7)

## Figure 1. Directed acyclic graph outlining assumptions of Mendelian Randomisation analysis

X

G1: IL-6

Self-harm

U1

IL-6

G2: CRP

CRP

U2

X

Notes: G: genetic instruments, U: unmeasured confounders, IL-6 Interleukin-6, CRP C-reactive protein. Dotted lines indicate that pathways between the instrument and outcome should only operate through the phenotype i.e. there are no additional pleiotropic effects. There were 2 genetic instruments for IL-6 and 46 for CRP.

## Table 1. Self-harm GWAS in UK Biobank: SNPs meeting suggestive significance (p=<5x10^-6^)

| SNP | CHR | Effect allele | Other allele | Effect allele frequency | OR | SE | P |
| --- | --- | --- | --- | --- | --- | --- | --- |
| rs375968703 | 16 | A | G | 0.76 | 0.98 | 1.03 | 8.10E-08 |
| rs73852526 | 4 | A | G | 0.83 | 1.00 | 1.03 | 1.70E-07 |
| rs17088101 | 6 | T | C | 1 | 1.00 | 1.05 | 3.20E-07 |
| rs543749957 | 5 | C | T | 1 | 1.00 | 1.05 | 4.80E-07 |
| rs10968364 | 9 | A | G | 0.89 | 1.02 | 1.06 | 4.90E-07 |
| rs1452348 | 9 | T | G | 0.89 | 1.07 | 1.04 | 5.00E-07 |
| rs114039181 | 5 | G | A | 0.99 | 1.07 | 1.04 | 5.30E-07 |
| rs12238130 | 9 | G | A | 0.91 | 1.01 | 1.04 | 5.30E-07 |
| rs79119373 | 9 | A | G | 0.89 | 0.95 | 1.04 | 6.00E-07 |
| rs8035597 | 15 | A | C | 0.68 | 1.08 | 1.04 | 6.20E-07 |
| rs76791169 | 9 | T | C | 0.89 | 1.08 | 1.04 | 6.50E-07 |
| 5:81347083_GC_G | 5 | GC | G | 1 | 1.02 | 1.03 | 7.20E-07 |
| rs181000387 | 5 | A | G | 0.99 | 1.05 | 1.07 | 7.40E-07 |
| rs10968363 | 9 | G | A | 0.89 | 1.03 | 1.03 | 7.70E-07 |
| rs80095685 | 9 | G | A | 0.89 | 0.97 | 1.03 | 8.50E-07 |
| rs12148067 | 15 | A | G | 0.69 | 1.00 | 1.03 | 8.70E-07 |
| rs72666653 | 1 | T | C | 0.9 | 1.19 | 1.08 | 1.00E-06 |
| rs187627524 | 1 | A | G | 0.99 | 1.01 | 1.05 | 1.10E-06 |
| rs5775181 | 1 | C | CA | 0.67 | 1.00 | 1.06 | 1.10E-06 |
| rs12567259 | 1 | A | T | 0.67 | 1.07 | 1.03 | 1.10E-06 |
| rs150574 | 1 | A | G | 0.71 | 1.04 | 1.05 | 1.20E-06 |
| rs215817 | 1 | A | C | 0.7 | 1.00 | 1.03 | 1.30E-06 |
| rs215810 | 1 | G | C | 0.7 | 0.97 | 1.04 | 1.30E-06 |
| rs215808 | 1 | T | G | 0.7 | 0.99 | 1.03 | 1.30E-06 |
| rs2497991 | 1 | A | G | 0.7 | 1.02 | 1.03 | 1.30E-06 |
| rs215857 | 1 | T | C | 0.7 | 1.05 | 1.03 | 1.30E-06 |
| rs184231091 | 1 | C | G | 0.99 | 0.98 | 1.03 | 1.30E-06 |
| rs9633290 | 1 | G | C | 0.68 | 1.03 | 1.05 | 1.30E-06 |
| rs9633294 | 1 | C | T | 0.68 | 1.05 | 1.05 | 1.30E-06 |
| rs9633295 | 1 | G | A | 0.68 | 1.06 | 1.05 | 1.30E-06 |
| rs138177472 | 3 | C | T | 1 | 1.01 | 1.03 | 1.30E-06 |
| rs215809 | 1 | T | C | 0.7 | 1.03 | 1.03 | 1.40E-06 |
| rs629121 | 1 | A | C | 0.7 | 1.03 | 1.04 | 1.40E-06 |
| rs2497990 | 1 | T | C | 0.7 | 0.97 | 1.03 | 1.40E-06 |
| rs215840 | 1 | A | G | 0.7 | 1.08 | 1.05 | 1.40E-06 |
| rs2208564 | 1 | A | T | 0.68 | 0.94 | 1.05 | 1.40E-06 |
| rs79360421 | 5 | T | G | 0.91 | 1.04 | 1.08 | 1.40E-06 |
| rs215811 | 1 | C | T | 0.7 | 0.93 | 1.05 | 1.50E-06 |
| rs215807 | 1 | C | T | 0.7 | 1.00 | 1.03 | 1.50E-06 |
| rs215842 | 1 | A | T | 0.7 | 0.93 | 1.08 | 1.50E-06 |
| rs12024899 | 1 | C | A | 0.68 | 0.93 | 1.08 | 1.50E-06 |
| rs150362834 | 3 | C | G | 1 | 1.07 | 1.05 | 1.50E-06 |
| rs578115096 | 7 | G | A | 1 | 1.03 | 1.05 | 1.50E-06 |
| rs215818 | 1 | A | C | 0.7 | 1.08 | 1.05 | 1.60E-06 |
| rs215816 | 1 | C | A | 0.7 | 1.08 | 1.05 | 1.60E-06 |
| rs215815 | 1 | G | A | 0.7 | 1.01 | 1.08 | 1.60E-06 |
| rs215814 | 1 | T | C | 0.7 | 0.91 | 1.06 | 1.60E-06 |
| rs215806 | 1 | C | T | 0.7 | 1.02 | 1.05 | 1.60E-06 |
| rs7533561 | 1 | G | A | 0.9 | 1.02 | 1.05 | 1.60E-06 |
| rs1923242 | 1 | C | T | 0.67 | 1.04 | 1.03 | 1.60E-06 |
| rs12683152 | 9 | T | A | 0.91 | 0.96 | 1.05 | 1.60E-06 |
| rs215853 | 1 | G | C | 0.7 | 1.02 | 1.05 | 1.70E-06 |
| rs28685835 | 1 | C | T | 0.68 | 1.02 | 1.05 | 1.70E-06 |
| rs150371835 | 1 | C | T | 0.99 | 1.01 | 1.05 | 1.70E-06 |
| rs78547615 | 14 | G | C | 0.95 | 1.00 | 1.03 | 1.70E-06 |
| rs215848 | 1 | A | G | 0.7 | 0.95 | 1.05 | 1.80E-06 |
| rs12030954 | 1 | G | A | 0.68 | 0.95 | 1.05 | 1.80E-06 |
| rs72969741 | 6 | C | T | 0.93 | 0.94 | 1.05 | 1.80E-06 |
| rs138986247 | 8 | G | A | 1 | 1.05 | 1.05 | 1.80E-06 |
| rs137894188 | 15 | C | T | 1 | 1.01 | 1.05 | 1.80E-06 |
| rs215851 | 1 | A | T | 0.7 | 1.01 | 1.05 | 1.90E-06 |
| rs58054723 | 1 | G | C | 0.91 | 1.01 | 1.03 | 1.90E-06 |
| rs146497750 | 1 | A | G | 0.99 | 1.02 | 1.05 | 1.90E-06 |
| rs617085 | 19 | G | C | 0.28 | 1.04 | 1.03 | 1.90E-06 |
| rs17408393 | 18 | A | G | 0.6 | 0.97 | 1.03 | 2.00E-06 |
| rs569337 | 19 | T | C | 0.28 | 1.04 | 1.03 | 2.00E-06 |
| rs681921 | 19 | G | T | 0.28 | 1.01 | 1.03 | 2.00E-06 |
| rs215813 | 1 | C | T | 0.7 | 1.07 | 1.05 | 2.10E-06 |
| rs149437164 | 15 | G | A | 1 | 0.95 | 1.06 | 2.10E-06 |
| rs12959099 | 18 | A | G | 0.56 | 1.02 | 1.07 | 2.10E-06 |
| rs4291980 | 18 | T | G | 0.57 | 0.98 | 1.03 | 2.20E-06 |
| rs4321259 | 18 | G | A | 0.57 | 0.99 | 1.07 | 2.20E-06 |
| rs62099230 | 18 | G | A | 0.6 | 0.99 | 1.07 | 2.20E-06 |
| rs12563453 | 1 | A | T | 0.67 | 0.99 | 1.07 | 2.30E-06 |
| rs34224300 | 1 | T | TA | 0.68 | 0.99 | 1.03 | 2.40E-06 |
| rs17487277 | 18 | C | G | 0.6 | 1.01 | 1.07 | 2.40E-06 |
| rs4803194 | 19 | C | T | 0.28 | 1.03 | 1.03 | 2.40E-06 |
| rs11578757 | 1 | G | T | 0.73 | 1.04 | 1.07 | 2.50E-06 |
| rs10489910 | 1 | G | C | 0.9 | 1.03 | 1.05 | 2.50E-06 |
| rs147478791 | 1 | C | T | 0.99 | 1.01 | 1.03 | 2.60E-06 |
| rs473878 | 19 | C | T | 0.28 | 1.04 | 1.05 | 2.60E-06 |
| rs497378 | 19 | C | A | 0.28 | 1.06 | 1.04 | 2.70E-06 |
| 1:56803312_CA_C | 1 | CA | C | 1 | 1.04 | 1.04 | 2.80E-06 |
| rs10403886 | 19 | T | C | 0.28 | 0.97 | 1.06 | 2.80E-06 |
| 18:50678953_GA_G | 18 | GA | G | 0.44 | 1.01 | 1.04 | 3.00E-06 |
| rs215819 | 1 | G | A | 0.7 | 1.04 | 1.04 | 3.10E-06 |
| rs76276668 | 1 | G | A | 0.91 | 1.02 | 1.04 | 3.10E-06 |
| rs12918742 | 16 | T | A | 0.92 | 0.97 | 1.06 | 3.10E-06 |
| rs72902949 | 2 | G | A | 0.57 | 0.92 | 1.04 | 3.20E-06 |
| rs12997689 | 2 | C | T | 0.57 | 0.96 | 1.07 | 3.20E-06 |
| rs141541070 | 15 | C | T | 1 | 1.02 | 1.05 | 3.20E-06 |
| rs1840748 | 19 | C | T | 0.5 | 1.07 | 1.06 | 3.20E-06 |
| rs1840746 | 19 | T | C | 0.5 | 0.99 | 1.07 | 3.20E-06 |
| rs599752 | 19 | C | T | 0.28 | 0.93 | 1.06 | 3.20E-06 |
| rs61177670 | 1 | C | CA | 0.47 | 0.90 | 1.06 | 3.30E-06 |
| rs12162359 | 2 | C | T | 0.57 | 0.97 | 1.07 | 3.30E-06 |
| rs2488647 | 10 | A | C | 1 | 0.94 | 1.04 | 3.30E-06 |
| rs4372758 | 18 | C | T | 0.44 | 0.86 | 1.07 | 3.30E-06 |
| rs631879 | 19 | A | G | 0.28 | 0.99 | 1.03 | 3.30E-06 |
| rs11210177 | 1 | A | G | 0.5 | 1.08 | 1.05 | 3.40E-06 |
| rs1852305 | 2 | G | A | 0.57 | 0.89 | 1.05 | 3.40E-06 |
| rs116917481 | 13 | C | T | 0.97 | 0.96 | 1.08 | 3.40E-06 |
| rs1840747 | 19 | T | G | 0.5 | 0.99 | 1.04 | 3.40E-06 |
| rs34589249 | 19 | A | AT | 0.5 | 0.93 | 1.03 | 3.40E-06 |
| rs12743241 | 1 | G | A | 0.74 | 0.97 | 1.03 | 3.50E-06 |
| rs13004465 | 2 | A | G | 0.57 | 1.03 | 1.07 | 3.50E-06 |
| rs139566208 | 15 | C | T | 1 | 0.96 | 1.04 | 3.50E-06 |
| rs476616 | 19 | G | A | 0.28 | 1.03 | 1.03 | 3.50E-06 |
| rs7349340 | 2 | A | G | 0.57 | 1.01 | 1.03 | 3.60E-06 |
| rs892359 | 2 | A | G | 0.57 | 0.97 | 1.03 | 3.60E-06 |
| rs116915066 | 6 | C | T | 0.99 | 0.97 | 1.06 | 3.60E-06 |
| rs72666659 | 1 | A | G | 0.91 | 0.97 | 1.06 | 3.70E-06 |
| rs72655522 | 4 | A | C | 0.99 | 0.90 | 1.05 | 3.70E-06 |
| rs74890462 | 8 | T | G | 1 | 0.98 | 1.03 | 3.70E-06 |
| rs4280369 | 19 | T | C | 0.5 | 0.98 | 1.03 | 3.70E-06 |
| rs11083499 | 19 | G | A | 0.5 | 1.00 | 1.06 | 3.70E-06 |
| rs10420169 | 19 | T | C | 0.48 | 0.89 | 1.07 | 3.70E-06 |
| rs10922324 | 1 | A | T | 0.81 | 0.96 | 1.03 | 3.80E-06 |
| rs12993899 | 2 | T | C | 0.57 | 1.00 | 1.03 | 3.80E-06 |
| rs183587781 | 15 | C | T | 0.99 | 0.84 | 1.08 | 3.80E-06 |
| rs11670486 | 19 | G | A | 0.5 | 1.30 | 1.09 | 3.80E-06 |
| rs11670520 | 19 | G | C | 0.5 | 1.00 | 1.04 | 3.80E-06 |
| rs10416823 | 19 | T | G | 0.5 | 0.89 | 1.07 | 3.80E-06 |
| rs6508842 | 19 | G | A | 0.49 | 0.98 | 1.03 | 3.80E-06 |
| rs10418645 | 19 | C | T | 0.48 | 1.07 | 1.06 | 3.80E-06 |
| rs10418648 | 19 | C | T | 0.48 | 1.07 | 1.05 | 3.80E-06 |
| rs600128 | 19 | G | C | 0.28 | 0.99 | 1.04 | 3.80E-06 |
| rs9892119 | 17 | T | A | 0.9 | 0.88 | 1.07 | 3.90E-06 |
| rs7259801 | 19 | G | A | 0.5 | 1.02 | 1.04 | 3.90E-06 |
| rs7248513 | 19 | A | G | 0.5 | 1.01 | 1.04 | 3.90E-06 |
| rs7248652 | 19 | C | T | 0.5 | 1.01 | 1.04 | 3.90E-06 |
| rs10853725 | 19 | C | G | 0.49 | 1.01 | 1.04 | 3.90E-06 |
| rs7258862 | 19 | C | T | 0.49 | 1.01 | 1.04 | 3.90E-06 |
| rs10424827 | 19 | C | T | 0.49 | 1.01 | 1.04 | 3.90E-06 |
| 1:73661163_GT_G | 1 | GT | G | 0.52 | 1.04 | 1.08 | 4.00E-06 |
| rs113062598 | 9 | T | A | 0.89 | 1.04 | 1.08 | 4.00E-06 |
| rs4343352 | 18 | T | C | 0.56 | 1.00 | 1.03 | 4.00E-06 |
| rs8104751 | 19 | C | T | 0.49 | 1.02 | 1.03 | 4.00E-06 |
| rs17701443 | 2 | G | A | 0.57 | 0.99 | 1.04 | 4.10E-06 |
| rs35828510 | 2 | C | G | 0.56 | 1.00 | 1.03 | 4.10E-06 |
| rs35536025 | 5 | T | TA | 0.59 | 1.09 | 1.05 | 4.10E-06 |
| rs143636724 | 8 | C | G | 1 | 1.01 | 1.05 | 4.10E-06 |
| rs7247801 | 19 | T | C | 0.49 | 0.96 | 1.07 | 4.10E-06 |
| rs151201760 | 19 | C | G | 1 | 0.99 | 1.05 | 4.10E-06 |
| rs6508843 | 19 | A | G | 0.49 | 0.96 | 1.05 | 4.20E-06 |
| rs10418986 | 19 | G | A | 0.48 | 0.98 | 1.04 | 4.20E-06 |
| rs11210187 | 1 | T | C | 0.52 | 1.00 | 1.07 | 4.30E-06 |
| rs576009941 | 4 | G | C | 0.99 | 0.98 | 1.03 | 4.30E-06 |
| rs1840745 | 19 | G | A | 0.5 | 0.98 | 1.03 | 4.30E-06 |
| rs4803186 | 19 | A | G | 0.49 | 0.98 | 1.03 | 4.30E-06 |
| rs35517564 | 19 | T | TA | 0.5 | 0.96 | 1.08 | 4.30E-06 |
| rs11880441 | 19 | T | C | 0.49 | 0.91 | 1.06 | 4.30E-06 |
| rs4803187 | 19 | C | A | 0.49 | 1.00 | 1.05 | 4.30E-06 |
| rs4650206 | 1 | T | C | 0.52 | 1.06 | 1.05 | 4.40E-06 |
| rs4074990 | 1 | C | G | 0.52 | 1.04 | 1.03 | 4.40E-06 |
| rs34313173 | 5 | T | TA | 0.55 | 1.00 | 1.03 | 4.40E-06 |
| rs71369719 | 17 | G | A | 1 | 1.00 | 1.06 | 4.40E-06 |
| rs11083500 | 19 | C | T | 0.5 | 0.98 | 1.03 | 4.40E-06 |
| rs36154890 | 19 | T | C | 0.5 | 0.98 | 1.03 | 4.40E-06 |
| rs4803185 | 19 | G | A | 0.5 | 0.94 | 1.05 | 4.40E-06 |
| rs11083502 | 19 | A | C | 0.49 | 1.07 | 1.06 | 4.40E-06 |
| rs12972850 | 19 | C | T | 0.49 | 1.03 | 1.06 | 4.40E-06 |
| rs8104990 | 19 | C | T | 0.5 | 1.07 | 1.04 | 4.40E-06 |
| rs4650205 | 1 | T | A | 0.52 | 0.94 | 1.05 | 4.50E-06 |
| 1:245602808_CT_C | 1 | CT | C | 0.98 | 1.03 | 1.02 | 4.50E-06 |
| rs1455853 | 5 | G | A | 0.01 | 1.04 | 1.03 | 4.50E-06 |
| rs3848623 | 19 | T | C | 0.5 | 0.98 | 1.03 | 4.50E-06 |
| rs11083501 | 19 | A | G | 0.49 | 0.93 | 1.05 | 4.50E-06 |
| rs215820 | 1 | C | T | 0.74 | 0.99 | 1.05 | 4.60E-06 |
| rs72666658 | 1 | G | A | 0.91 | 1.09 | 1.07 | 4.60E-06 |
| rs4261072 | 1 | A | T | 0.52 | 1.03 | 1.06 | 4.60E-06 |
| rs13008827 | 2 | C | A | 0.56 | 1.06 | 1.06 | 4.60E-06 |
| rs12985447 | 19 | C | T | 0.5 | 1.05 | 1.06 | 4.60E-06 |
| rs8104899 | 19 | C | T | 0.5 | 1.03 | 1.06 | 4.60E-06 |
| rs8105865 | 19 | T | G | 0.5 | 1.05 | 1.07 | 4.60E-06 |
| rs770411385 | 19 | GGCTCT | G | 0.49 | 0.99 | 1.06 | 4.60E-06 |
| rs10417436 | 19 | G | A | 0.5 | 1.02 | 1.06 | 4.60E-06 |
| rs4296324 | 18 | C | G | 0.57 | 1.01 | 1.05 | 4.70E-06 |
| rs7229097 | 18 | G | A | 0.44 | 1.01 | 1.05 | 4.70E-06 |
| rs4803188 | 19 | C | A | 0.49 | 1.00 | 1.05 | 4.70E-06 |
| rs769369572 | 19 | TGTCC | T | 0.18 | 1.06 | 1.08 | 4.80E-06 |
| rs10424632 | 19 | C | T | 0.49 | 0.95 | 1.11 | 4.80E-06 |
| rs12092858 | 1 | A | T | 0.52 | 1.06 | 1.06 | 4.90E-06 |
| rs116860797 | 8 | G | A | 0.98 | 1.01 | 1.05 | 4.90E-06 |
| rs138828454 | 14 | A | G | 0.99 | 1.11 | 1.07 | 4.90E-06 |
| rs12971842 | 19 | T | C | 0.5 | 1.01 | 1.05 | 4.90E-06 |
| rs10890020 | 1 | A | G | 0.52 | 1.06 | 1.06 | 5.00E-06 |
| rs10890021 | 1 | T | A | 0.52 | 1.07 | 1.06 | 5.00E-06 |
| rs12136984 | 1 | T | G | 0.51 | 1.03 | 1.04 | 5.00E-06 |
| rs145247880 | 2 | C | T | 1 | 1.06 | 1.06 | 5.00E-06 |
| rs527329038 | 5 | C | T | 1 | 1.05 | 1.06 | 5.00E-06 |
| rs12972978 | 19 | T | C | 0.5 | 1.05 | 1.06 | 5.00E-06 |
| rs472104 | 19 | G | A | 0.28 | 1.01 | 1.07 | 5.00E-06 |

## Table 2. Suicide attempt GWAS: SNPs meeting suggestive significance (p=<5x10^-6^)

| SNP | CHR | Effect allele | Other allele | Effect allele frequency | OR | SE | P |
| --- | --- | --- | --- | --- | --- | --- | --- |
| rs10178415 | 2 | T | C | 0.64 | 1.50 | 1.09 | 7.10E-07 |
| rs4589382 | 12 | C | G | 0.25 | 1.62 | 1.10 | 9.70E-07 |
| rs760157231 | 2 | CACA | C | 0.44 | 1.49 | 1.09 | 1.40E-06 |
| rs67908106 | 2 | A | G | 0.64 | 1.48 | 1.09 | 1.70E-06 |
| rs28647531 | 4 | G | A | 0.90 | 0.54 | 1.14 | 1.90E-06 |
| rs10517752 | 4 | A | G | 0.90 | 0.54 | 1.14 | 2.60E-06 |
| rs6538182 | 12 | G | A | 0.62 | 0.68 | 1.09 | 2.70E-06 |
| rs9668297 | 12 | A | G | 0.62 | 0.68 | 1.09 | 2.90E-06 |
| rs35780879 | 12 | A | T | 0.62 | 0.68 | 1.09 | 3.00E-06 |
| rs34691336 | 12 | G | A | 0.62 | 0.68 | 1.09 | 3.10E-06 |
| rs67121530 | 12 | C | T | 0.61 | 0.68 | 1.09 | 3.20E-06 |
| rs11105336 | 12 | C | A | 0.62 | 0.68 | 1.09 | 3.30E-06 |
| rs4533086 | 12 | T | G | 0.61 | 0.69 | 1.09 | 3.70E-06 |
| rs35450770 | 8 | C | G | 0.88 | 0.57 | 1.13 | 4.00E-06 |
| rs67595273 | 12 | A | C | 0.18 | 1.62 | 1.11 | 4.10E-06 |

## Figure 2. A) Manhatten plot and b) QQ plot of suicide attempt GWAS in UK Biobank

## A

##
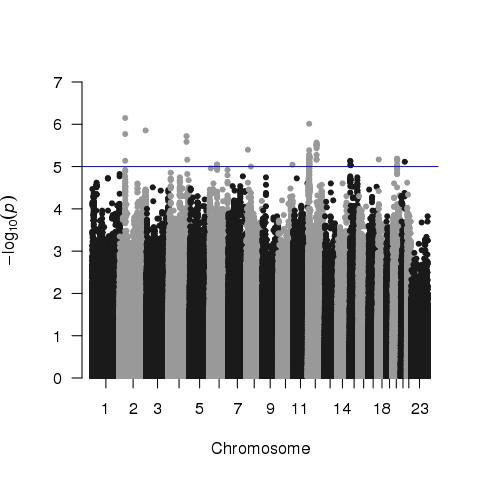


## B


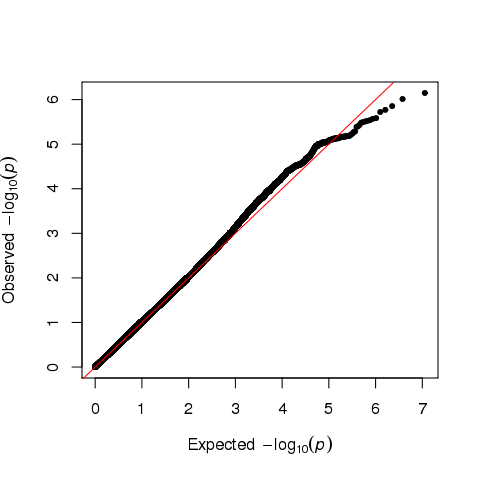


## Table 3. CRP instruments

| SNP | CHR | BP | Effect allele | Other allele | Beta | SE | P |
| --- | --- | --- | --- | --- | --- | --- | --- |
| rs75460349 | 1 | 27180088 | a | c | 0.086 | 0.014 | 4.88E-10 |
| rs4660293 | 1 | 40028180 | a | g | -0.038 | 0.005 | 1.96E-14 |
| rs4311928 | 1 | 66119752 | t | c | 0.103 | 0.004 | 0.00E+00 |
| rs469773 | 1 | 91530259 | t | c | -0.034 | 0.005 | 2.99E-11 |
| rs56023096 | 1 | 154291416 | t | c | -0.045 | 0.006 | 1.12E-13 |
| rs2228145 | 1 | 154426970 | a | c | 0.090 | 0.004 | 0.00E+00 |
| rs115804053 | 1 | 159345035 | a | g | -0.100 | 0.016 | 1.16E-09 |
| rs115615753 | 1 | 159359030 | t | c | -0.081 | 0.013 | 9.17E-11 |
| rs144970957 | 1 | 159514964 | t | c | 0.161 | 0.015 | 3.04E-28 |
| rs2592887 | 1 | 159652939 | t | c | -0.158 | 0.004 | 0.00E+00 |
| rs4925546 | 1 | 247602968 | a | g | -0.043 | 0.004 | 2.79E-24 |
| rs10832027 | 11 | 13357183 | a | g | 0.027 | 0.004 | 2.17E-10 |
| rs4752829 | 11 | 47396654 | a | g | -0.026 | 0.005 | 9.52E-09 |
| rs1582763 | 11 | 60021948 | a | g | -0.026 | 0.004 | 8.28E-10 |
| rs12813389 | 12 | 95913562 | a | t | -0.033 | 0.004 | 1.02E-15 |
| rs7135240 | 12 | 103535020 | a | g | -0.025 | 0.004 | 6.80E-10 |
| rs2393794 | 12 | 121378976 | t | c | -0.034 | 0.006 | 2.72E-09 |
| rs7979473 | 12 | 121420260 | a | g | -0.147 | 0.004 | 0.00E+00 |
| rs2239222 | 14 | 73011885 | a | g | -0.038 | 0.004 | 7.08E-18 |
| rs112635299 | 14 | 94838142 | t | g | -0.107 | 0.017 | 2.22E-10 |
| rs1189402 | 15 | 53728154 | a | g | 0.025 | 0.004 | 2.64E-09 |
| rs340005 | 15 | 60878030 | a | g | 0.036 | 0.004 | 3.12E-17 |
| rs116971887 | 16 | 51170026 | t | g | -0.113 | 0.012 | 2.33E-20 |
| rs2110840 | 16 | 51410819 | a | c | -0.059 | 0.007 | 5.57E-15 |
| rs8047395 | 16 | 53798523 | a | g | 0.026 | 0.004 | 8.11E-10 |
| rs2384955 | 17 | 72695167 | t | c | -0.038 | 0.006 | 7.59E-11 |
| rs2847286 | 18 | 12817815 | a | g | -0.025 | 0.004 | 1.97E-09 |
| rs2972558 | 19 | 45356141 | t | c | -0.039 | 0.005 | 4.51E-15 |
| rs429358 | 19 | 45411941 | t | c | 0.247 | 0.006 | 0.00E+00 |
| rs13022337 | 2 | 632609 | a | g | -0.032 | 0.005 | 4.35E-09 |
| rs1260326 | 2 | 27730940 | t | c | 0.069 | 0.004 | 0.00E+00 |
| rs1115282 | 2 | 102890970 | t | c | 0.024 | 0.004 | 2.29E-09 |
| rs6734238 | 2 | 113841030 | a | g | -0.046 | 0.004 | 7.46E-29 |
| rs1800961 | 20 | 43042364 | t | c | -0.099 | 0.013 | 2.38E-15 |
| rs4817984 | 21 | 40465066 | a | c | -0.039 | 0.005 | 8.86E-17 |
| rs4821816 | 22 | 39113134 | a | g | -0.028 | 0.004 | 3.55E-10 |
| rs10049413 | 3 | 49892896 | a | g | -0.028 | 0.004 | 3.69E-10 |
| rs7356034 | 3 | 170732599 | a | g | 0.027 | 0.004 | 2.59E-09 |
| rs34471628 | 5 | 172196752 | a | g | -0.077 | 0.012 | 3.71E-11 |
| rs34039593 | 6 | 32570311 | t | g | -0.045 | 0.007 | 2.97E-09 |
| rs1490384 | 6 | 126851160 | t | c | -0.026 | 0.004 | 2.28E-10 |
| rs13241897 | 7 | 22745351 | a | g | 0.031 | 0.004 | 1.10E-13 |
| rs12673996 | 7 | 22833400 | t | c | -0.028 | 0.005 | 1.35E-08 |
| rs7797566 | 7 | 72896395 | a | c | 0.050 | 0.006 | 6.35E-15 |
| rs7012637 | 8 | 9173209 | a | g | 0.054 | 0.004 | 1.99E-36 |
| rs6987444 | 8 | 117076315 | a | g | 0.034 | 0.004 | 3.17E-16 |
| rs10956251 | 8 | 126513330 | t | c | 0.038 | 0.006 | 2.39E-09 |
| rs505922 | 9 | 136149229 | t | c | -0.026 | 0.004 | 9.58E-10 |

## Table 4. IL-6 instruments

| SNP | Chr | Gene | Allele1 | Allele2 | Beta | SE | P |
| --- | --- | --- | --- | --- | --- | --- | --- |
| rs643434 | 9 | ABO | a | g | -0.257 | 0.017 | 1.44E-48 |
| rs56383622 | 1 | IL6R | a | g | -0.231 | 0.024 | 1.34E-22 |

## Table 5. Additional IL-6 instruments for post-hoc analysis

| SNP | Chr | Gene | Allele1 | Allele2 | Beta | SE | P |
| --- | --- | --- | --- | --- | --- | --- | --- |
| rs1542176 | 2 | None | t | c | 0.0044 | 0.016 | 0.7863 |
| rs6743376 | 2 | IL1F10 | a | c | 0.0207 | 0.0177 | 0.243 |
| rs7529229 | 1 | IL6R | t | c | -0.2054 | 0.0212 | 4.154E-22 |

## CRP plots: two sample MR with varying methods


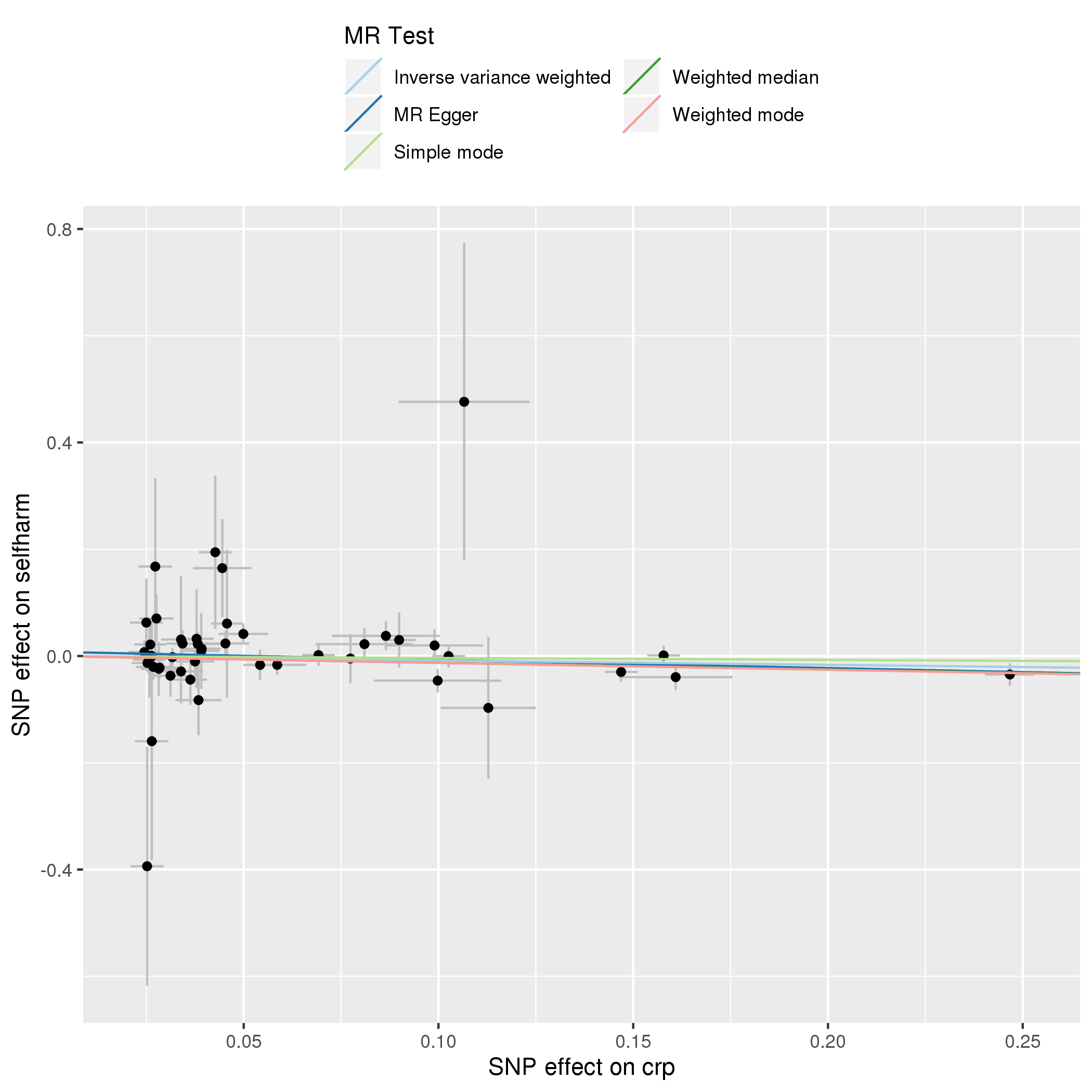


## Funnel plot of CRP SNPs
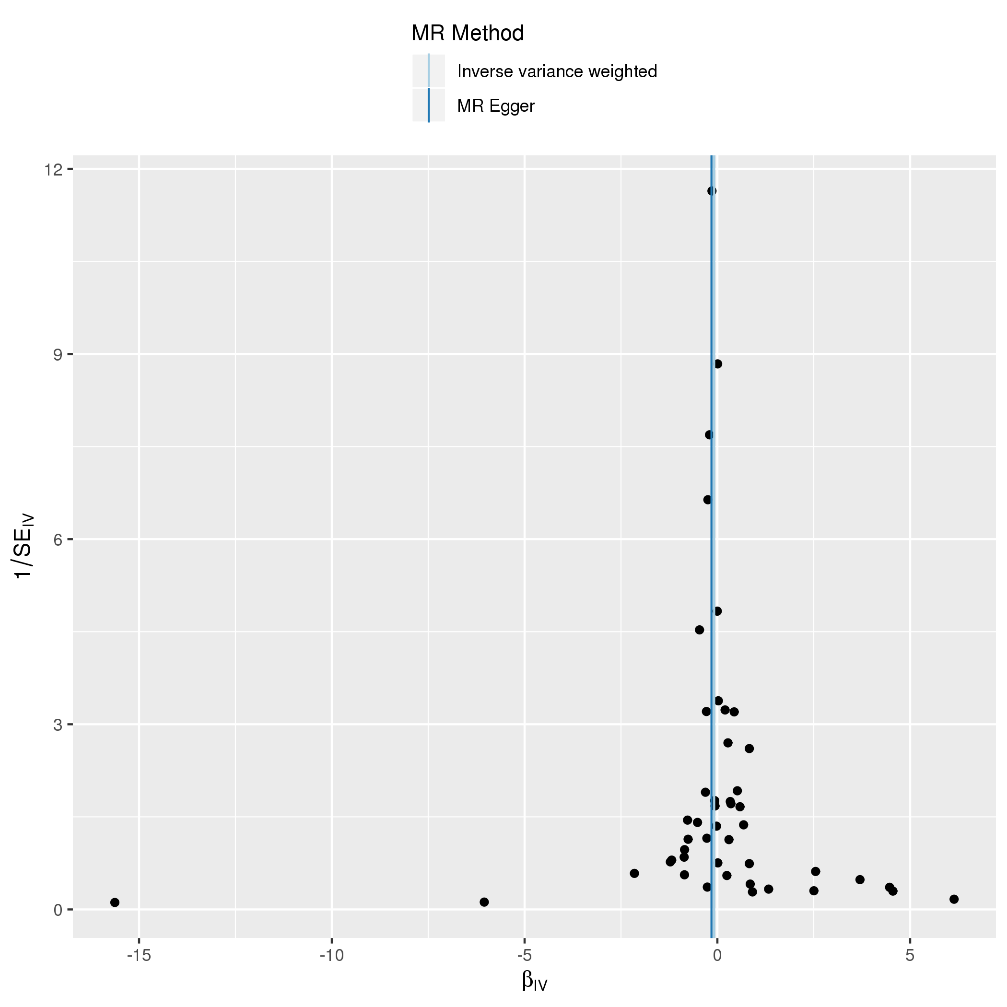


## Single SNP plots- CRP


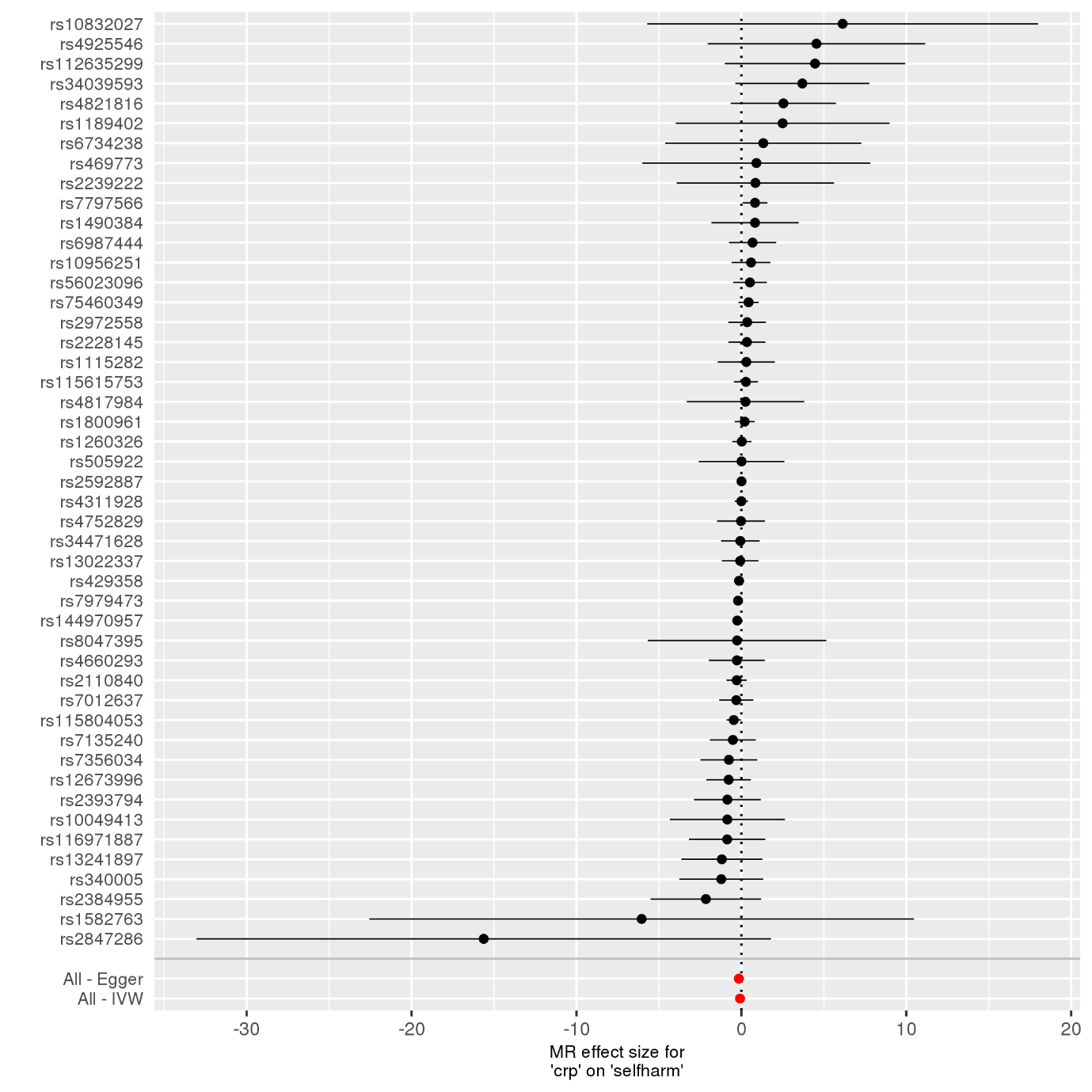


## Leave-one-out analysis CRP: is a single SNP driving the association?


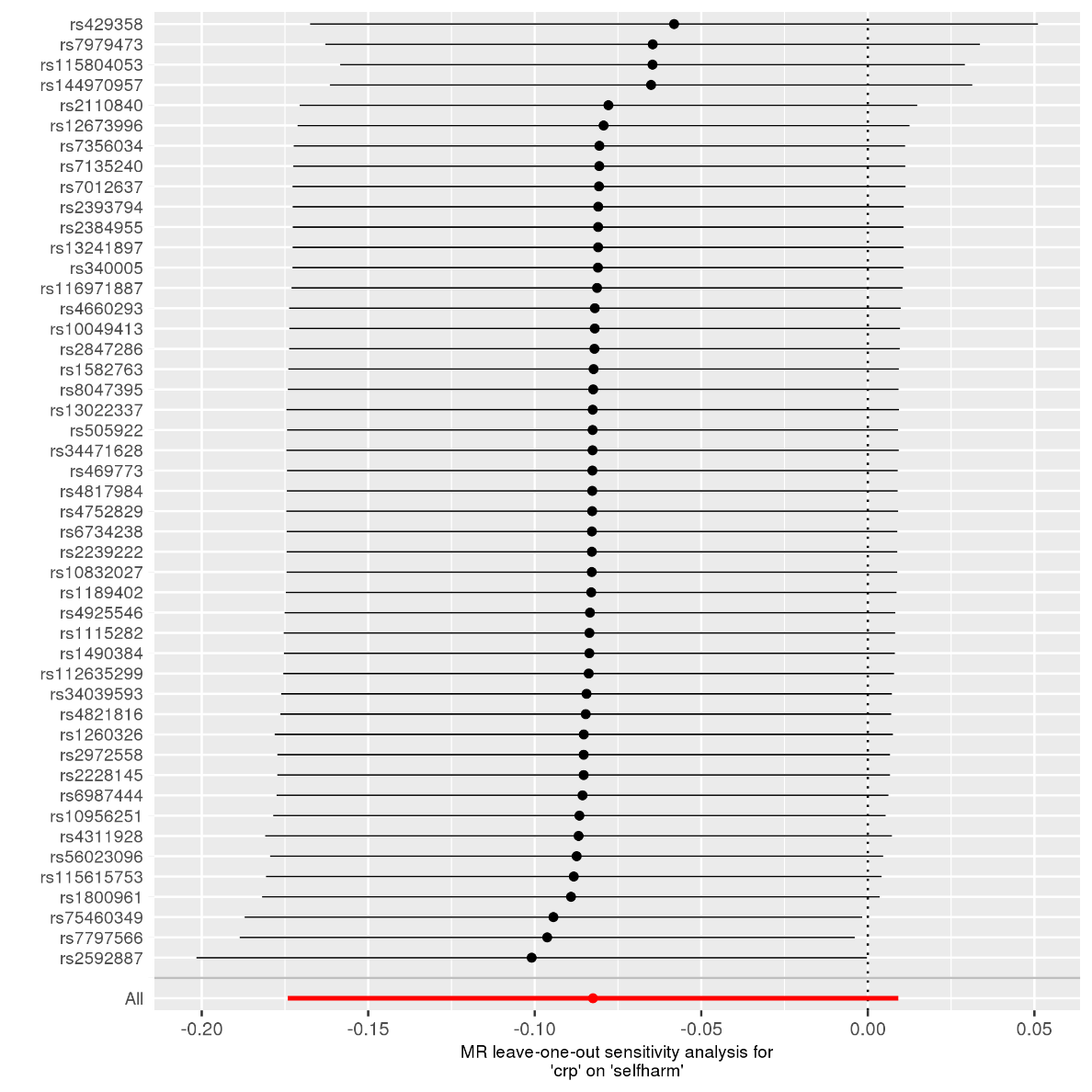


1. Boyd A, Golding J, Macleod J, Lawlor DA, Fraser A, Henderson J, et al. Cohort profile: the ‘children of the 90s’—the index offspring of the Avon Longitudinal Study of Parents and Children. International journal of epidemiology. 2013;42(1):111-27.

2. Fraser A, Macdonald-Wallis C, Tilling K, Boyd A, Golding J, Davey Smith G, et al. Cohort profile: the Avon Longitudinal Study of Parents and Children: ALSPAC mothers cohort. International journal of epidemiology. 2012;42(1):97-110.

3. Khandaker GM, Pearson RM, Zammit S, Lewis G, Jones PB. Association of serum interleukin 6 and C-reactive protein in childhood with depression and psychosis in young adult life: a population-based longitudinal study. JAMA psychiatry. 2014;71(10):1121-8.

4. Mitchell R, Hemani G, Dudding T, Paternoster L. UK biobank genetic data: MRC-IEU quality control, Version 1. University of Bristol. 2017.

5. Bycroft C, Freeman C, Petkova D, Band G, Elliott LT, Sharp K, et al. The UK Biobank resource with deep phenotyping and genomic data. Nature. 2018;562(7726):203.

6. Howie B, Marchini J, Stephens M. Genotype imputation with thousands of genomes. G3: Genes, Genomes, Genetics. 2011;1(6):457-70.

7. Ruth Mitchell GH, Tom Dudding, Laura Corbin, Sean Harrison, Lavinia Paternoster. UK Biobank Genetic Data: MRC-IEU Quality Control, version 2. 2019.

8. Khandaker GM, Zuber V, Rees JMB, Carvalho L, Mason AM, Foley CN, et al. Shared mechanisms between coronary heart disease and depression: findings from a large UK general population-based cohort. Molecular Psychiatry. 2019.
